# FINDING FULFILLMENT IN NEW BEGINNINGS: EXPLORING LIFE SATISFACTION AMONG RESETTLED BHUTANESE OLDER ADULTS IN OHIO

**DOI:** 10.1093/geroni/igae098.1731

**Published:** 2024-12-31

**Authors:** Isha Karmacharya, Saruna Ghimire

**Affiliations:** Miami University, Oxford, Ohio, United States; Miami University, Oxford, Ohio, United States

## Abstract

**Background:**

The decision for refugees to flee their home country is often driven by persecution, conflict, or other dire circumstances, leading to a significant disruption in their lives. The US, along with the state of Ohio, has provided a safety net for a significant number of Bhutanese refugees. However, resettled refugees, particularly older adults, face significant challenges in adjusting to their new environment. This study aims to assess the life satisfaction of resettled Bhutanese older adults in Ohio, USA, and to identify the factors influencing their life satisfaction.

**Methods:**

Through snowball sampling facilitated by local Bhutanese organizations, a survey was conducted among 276 resettled Bhutanese individuals aged 55 and above in four major Ohio cities (Columbus, Akron, Cleveland, and Cincinnati). Life satisfaction was assessed using the widely popular and validated Satisfaction with Life Scale, and binary logistic regression was employed to model the factors associated with life satisfaction.

**Results:**

One in ten participants reported dissatisfaction with their lives. Participants experiencing depressive symptoms had higher odds of life dissatisfaction (OR:9.02, 95% CI: 2.28-35.77, p=0.002), whereas those with greater social support (OR: 0.89, 95% CI: 0.85-0.94, p<0.0001) and resilience (OR:0.92, 95% CI: 0.88-0.97, p<0.002) had lower odds.

**Conclusion:**

Despite enduring significant challenges throughout their journey from displacement and refugee camps to resettlement in the USA, a notable proportion of participants expressed satisfaction with their lives. However, future studies should delve into their life transitions before and after resettlement to gain a deeper understanding of how the resettlement process shapes their life satisfaction.

